# Longitudinal clinical and radiographic evaluation reveals interleukin-6 as an indicator of persistent pulmonary injury in COVID-19

**DOI:** 10.7150/ijms.49728

**Published:** 2021-01-01

**Authors:** Baolin Liao, Zhipeng Liu, Libo Tang, Linghua Li, Qingxin Gan, Haiyan Shi, Qian Jiao, Yujuan Guan, Min Xie, Xi He, Han Zhao, Weilie Chen, Yanxia Liu, Liya Li, Yaping Wang, Yi Cao, Yaling Shi, Yongyin Li, Chunliang Lei

**Affiliations:** 1 Guangzhou Eighth People's Hospital, Guangzhou Medical University, Guangzhou, China; 2 Department of Infectious Diseases, Nanfang Hospital, Southern Medical University, Guangzhou, China

**Keywords:** COVID-19, interleukin-6, pulmonary injury, chest CT, sequential monitoring

## Abstract

**Rationale**: Previous studies of coronavirus disease 2019 (COVID-19) were mainly focused on cross-sectional analysis. In this study, we sought to evaluate the dynamic changes of immunological and radiographic features, and the association with the outcome of pulmonary lesions in COVID-19 patients.

**Methods**: Peripheral blood samples and radiographic data were collected longitudinally for up to 8 weeks from 158 laboratory-confirmed COVID-19 patients. The chest computed tomography (CT) scans were scored based on a semi-quantification assessment according to the extent of pulmonary abnormalities; the temporal change of the immunological and radiographic features was analyzed.

**Results:** Compared with mild and moderate patients, severe patients had significantly decreased counts of lymphocytes, CD4^+^ T cells, CD8^+^ T cells, and CD19^+^ B cells but dramatically elevated counts of neutrophils and levels of interleukin (IL)-6. Sequential monitoring showed a sustained increase in lymphocytes counts and significantly decreased levels of IL-6 in severe patients during the disease course. Notably, patients with persistent pulmonary lesions (CT score ≥ 5 in week 8) showed high levels of IL-6 during the follow-up period, compared with those with recovery lesions (CT score < 5 in week 8). More importantly, the peak expression of IL-6 prior to the aggravated lung injury was mainly found in patients with persistent lesions, and multivariate analysis showed that IL-6 level upon admission was an independent factor associated with the persistent pulmonary injury.

**Conclusion:** Prolonged elevation of IL-6 is associated with persistent pulmonary lesions in COVID-19 patients. Sequential monitoring and timely intervention of IL-6 may favor the clinical management of COVID-19.

## Introduction

Coronavirus disease 2019 (COVID-19) is a viral infection caused by severe acute respiratory syndrome coronavirus 2 (SARS-CoV-2), a novel β-coronavirus firstly identified during an outbreak of respiratory illness cases in Wuhan City, China 1. Unfortunately, the SARS-COV-2 virus spread worldwide and led to a pandemic of an extremely spreadable and potentially fatal disease. Currently, COVID-19 has become a public health issue of great concern 2. From the clinical observation, dry cough, sore throat, and fever are common symptoms in SARS-CoV-2 infected patients, and the majority of patients undergo spontaneous regression. However, some subjects developed various fatal complications, including organ failure, septic shock, severe pneumonia, and acute respiratory distress syndrome (ARDS) 3, which suggested that dysregulated immune responses involved in the development of SARS-CoV-2 infection.

Similar to abnormalities observed in severe acute respiratory syndrome coronavirus (SARS) and middle-east respiratory syndrome coronavirus (MERS) infections 4,5, lymphopenia and cytokine release syndrome are also essential features in patients with severe SARS-CoV-2 infection 6-9. Previous studies have shown that elevated levels of proinflammatory cytokines, such as TNF-α, IL-6, and IL-8, are associated with severe pulmonary injury and adverse outcomes of SARS and MERS infections 10,11. Indeed, the robust chronic inflammation induced in the latter phase of highly pathogenic coronavirus infections is the leading cause of ARDS-mediated lethal outcome 4. Recent findings reported increases in serum inflammatory cytokine (e.g., IL-2R, IL-6, IL-8, IL-10) in severe COVID-19 patients 9. Moreover, elevated levels of the inflammatory indicator IL-6 in the blood have been reported to be predictive of a fatal outcome in COVID-19 patients 12. Thus, inflammatory cytokines, especially IL-6, may play a vital role in the immunopathogenesis of SARS-COV-2 infection. Nevertheless, the evidence is far from robust and sufficient. Therefore, dynamic changes in immunological features, especially its association with pulmonary lesions, are urgently required.

With the help of a longitudinal cohort of COVID-19 patients who were followed for more than 8 weeks, this retrospective, single-center study aimed to investigate the dynamic changes of immunological and radiographic features, as well as the associations with the severity and outcome of pulmonary lesions in COVID-19 patients.

## Materials and Methods

### Study participants and clinical classification of COVID-19

A total of 158 patients with COVID-19 were recruited from January 22, 2020 to April 10, 2020 in Guangzhou Eighth People's Hospital, a designated hospital for the treatment of patients with COVID-19 in Guangzhou. All patients were confirmed with SARS-Cov-2 infection by nucleic acid detection. Patients less than 16 years old and without epidemiological, clinical, or immunological data were excluded. In addition, 25 healthy volunteers were enrolled as control. According to the Guideline for the Diagnosis and Treatment of New Coronavirus Pneumonia (7th edition, in Chinese) 13, COVID-19 patients were classified as follows: Mild patients (n = 14): mild symptoms and absence of radiological findings of pneumonia; Moderate patients (n = 110): fever or respiratory tract symptoms with radiological findings of pneumonia; Severe patients (n = 31): meeting any following condition: (1) polypnea and respiratory rate ≥ 30 times/minute; (2) oxygen saturation ≤ 93% at rest state; (3) PaO_2_/FIO_2_ ratio ≤ 300; (4) image manifestation indicates that pulmonary lesions deteriorate more than 50% within 24-48 hours. Critical ill patients (n = 3): meeting any following condition: (1) respiratory failure and require mechanical ventilation; (2) shock; (3) concomitant with other organ failure and require ICU treatment. In this study, critical ill patients were incorporated into the severe group due to the limited numbers; therefore, 3 groups were designated. This study was performed in accordance with Good Clinical Practice and the Declaration of Helsinki principles for ethical research. The study protocol was approved by the Ethics Committee of Guangzhou Eighth People's Hospital (Ethics number 202002135). Written informed consent was obtained from patients involved before enrollment when data were collected retrospectively.

### Clinical data collection

The clinical data were collected from electronic medical records, including demographic data, medical history, exposure history, symptoms, comorbidities, serum biochemical test (i.e., liver and renal function, creatine kinase, and electrolytes), coagulation profile. Chest computed tomographic (CT) scans, treatment measures (i.e., antiviral therapy, corticosteroid therapy, respiratory support, and kidney replacement therapy), and outcomes were also collected. The throat swab was tested to exclude evidence of other viral infections, including influenza, respiratory syncytial virus, avian influenza, parainfluenza, and adenovirus.

### Definitions of the clinical period

The time of disease onset was defined as the day when the symptom was noticed. The data regarding immune cell populations and cytokines were collected at weeks 2, 4, 6, and 8 after the onset of disease symptoms, respectively. As the various intervals of examinations among patients, data unavailable from the aforementioned time points were replaced by the primary data that closed to the indicated time point. Virus shedding period (VSP) was defined as the period between positive SARS-CoV-2 nucleic acid detection and negative SARS-CoV-2 (two consecutive undetectable SARS-CoV-2 nucleic acid tests after 48-hour interval); Length of hospitalization (LOH) was defined as the days of hospital stay; Incubation period (IP) was defined as the period between the date of exposure to the virus according to the contact history and the date of onset.

### CT imaging interpretation and score

Consecutive CT images were available from 110 patients as of the final observation time point. Two thoracic radiologists (more than 10 years of experience) blinded to the clinical data reviewed the CT images independently and resolved discrepancies by consensus. According to the CT manifestations available from the final observation time point, the presence or absence of the following image features was recorded: ground-glass opacities (GGO), consolidation, mixed GGO and consolidation, traction bronchiectasis, bronchial wall thickening, reticulation, subpleural bands, vascular enlargement, and lesion distribution. The detailed definitions of the above features were as previously described 14. Each lung was divided into three zones: upper (above the carina), middle (below the carina up to the inferior pulmonary vein), and lower (below the inferior pulmonary vein) zones 15. A semi-quantitative score was assigned for each lung zone according to a recent publication regarding chest CT findings of COVID-19 16. Briefly, score 0, 0% involvement; score 1, less than 25% involvement; score 2, 25% to less than 50% involvement; score 3, 50% to less than 75% involvement; and score 4, 75% or greater involvement. Summation of score from all 6 lung zones provided overall CT score (maximal CT score, 24). The CT involvement score < 5 was defined as recovery lesions, while score ≥ 5 was defined as persistent lesions.

### Real-time reverse transcription PCR (RT-PCR) assay for SARS-CoV-2

The throat swab was collected, and a test was performed to detect SARS-CoV-2 as previously described 17. In brief, viral RNA was extracted with the Nucleic Acid Isolation Kit (Da An Gene Corporation Ltd., China) following the guidelines. RT-PCR was performed using an RNA Detection Kit for SARS-CoV-2 (Da An Gene Corporation Ltd., China). Two PCR primer and probe sets, which target ORF1ab (FAM reporter) and N (VIC reporter) genes separately, were added in the same reaction tube. Positive and negative controls were included for each batch of detection. Samples were considered viral positive when either or both set(s) gave a reliable signal(s). The receiver operating characteristic (ROC) curve method was used to determine the internal standard reference cycle threshold (Ct) value, which was determined as 40. If the Ct value was ≤ 40, the sample was considered positive; if the value was > 40, the sample was negative.

### Serum cytokines measurement

The levels of serum IL-2, IL-4, IL-6, IL-10, TNF-α, and IFN-γ were quantitated by Cytometric Bead Array (CBA) Th1/Th2 kit (Weimi BioTech) in accordance with the manufacturer's instructions.

### Detection of immune cell subsets

The proportions of neutrophils, lymphocytes, monocytes, CD4^+^ T, CD8^+^ T, CD19^+^ B, NK (CD3^-^CD56^+^), and NKT (CD3^+^CD56^+^) cell were analyzed by flow cytometry. The absolute counts of immune cells were measured by using BD TruCount Absolute-Count Tubes (BD340334). The following antibodies (CD3-FITC, CD8-APC-Cy7, CD4-PE-Cy7, CD45-Percp-Cy5.5, CD56-PE, and CD19-APC) were used, and all reagents were purchased from BD Biosciences. All samples were detected by a BD FACS Canto II flow cytometry system and analyzed with the BD FACS Diva software.

### Statistical analysis

The distribution normality was assessed by the KolmogorovSmirnov test. Normally distributed data were expressed as mean ± SD and compared by one-way ANOVA. Non-normally distributed data were expressed as median with interquartile range (IQR), and Kruskal-Wallis H was used for multiple comparisons. Categorical data were presented as number (%) and analyzed by Chi-square or Fisher's exact test. Partial correlation or Spearman's correlation coefficients was used to calculate the correlations. Univariable logistics regression were used to identify factors associated with the severity of COVID-19 and the outcome of pulmonary lesions, variables with *P* value < 0.05 were included for multivariable analysis, the odds ratio (OR) along with the 95% confidence interval (CI) were reported. The area under the curve (AUC) and the 95% CI of the ROC curve was computed to evaluate the sensitivity and specificity of each parameter in predicting the severity of COVID-19 and the outcome of pulmonary lesions. The optimal cut-off points were determined by Youden's index. Statistical analysis was performed using the SPSS statistical software package for Microsoft Windows (SPSS, version 21, Chicago, IL). All the tests were two-sided, and a *P* value < 0.05 was considered statistically significant.

## Results

### The demographic and clinical characteristics of patients with COVID-19

**Figure 1:**
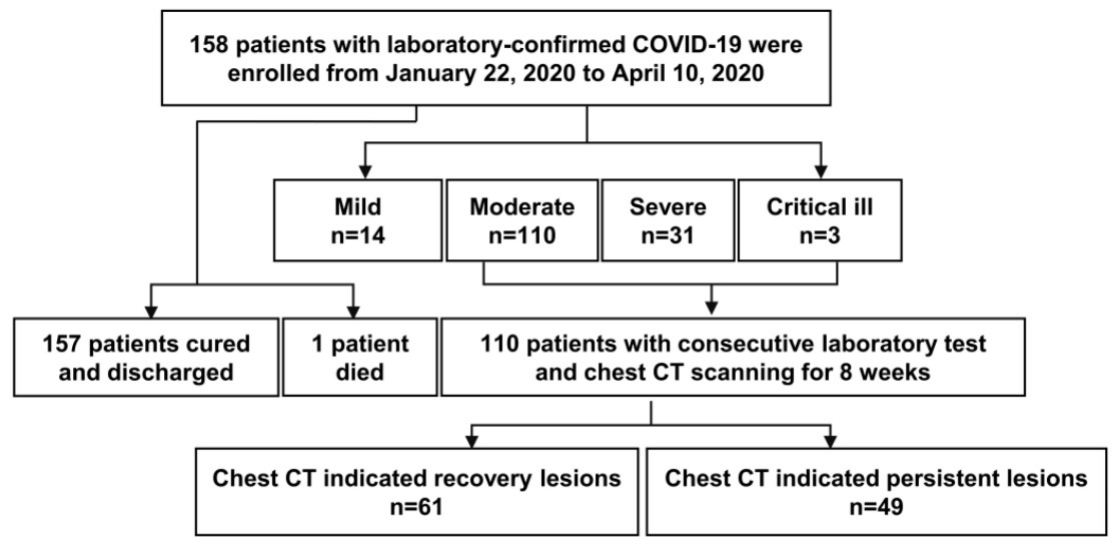
A flow diagram of patients recruited in this study.

The study population included 158 hospitalized patients with SARS-CoV-2 nucleic acid RT-PCR tests positive (Figure 1). Among them, the median incubation period was 6.0 (1.3 - 10.0) days, and 88 (57.1%) were men with a mean age of 48.0 ± 17.7 years old. Mild, moderate and severe (including critically ill) patients accounted for 8.9% (n = 14), 69.6% (n = 110), 21.5% (n = 34) patients, respectively. Patients of the severe group were significantly older (severe vs. moderate vs. mild: 62.7 ± 14.2 vs. 46.0 ± 15.8 vs. 28.1 ± 11.9, *P* < 0.001), and more inclined to have comorbidities and clinical symptoms, including fever, fatigue, diarrhea, polypnea, and anorexia. The median LOH was 31.0 (22.5 - 35.0) days in severe patients, 21.0 (14.0 - 28.0) days in moderate patients, 11.0 (7.0 - 19.0) days in mild patients. Besides, the virus shedding period was significantly delayed in severe patients (Table 1).

### Dynamic change of immune cell subpopulations in COVID-19 patients

**Figure 2:**
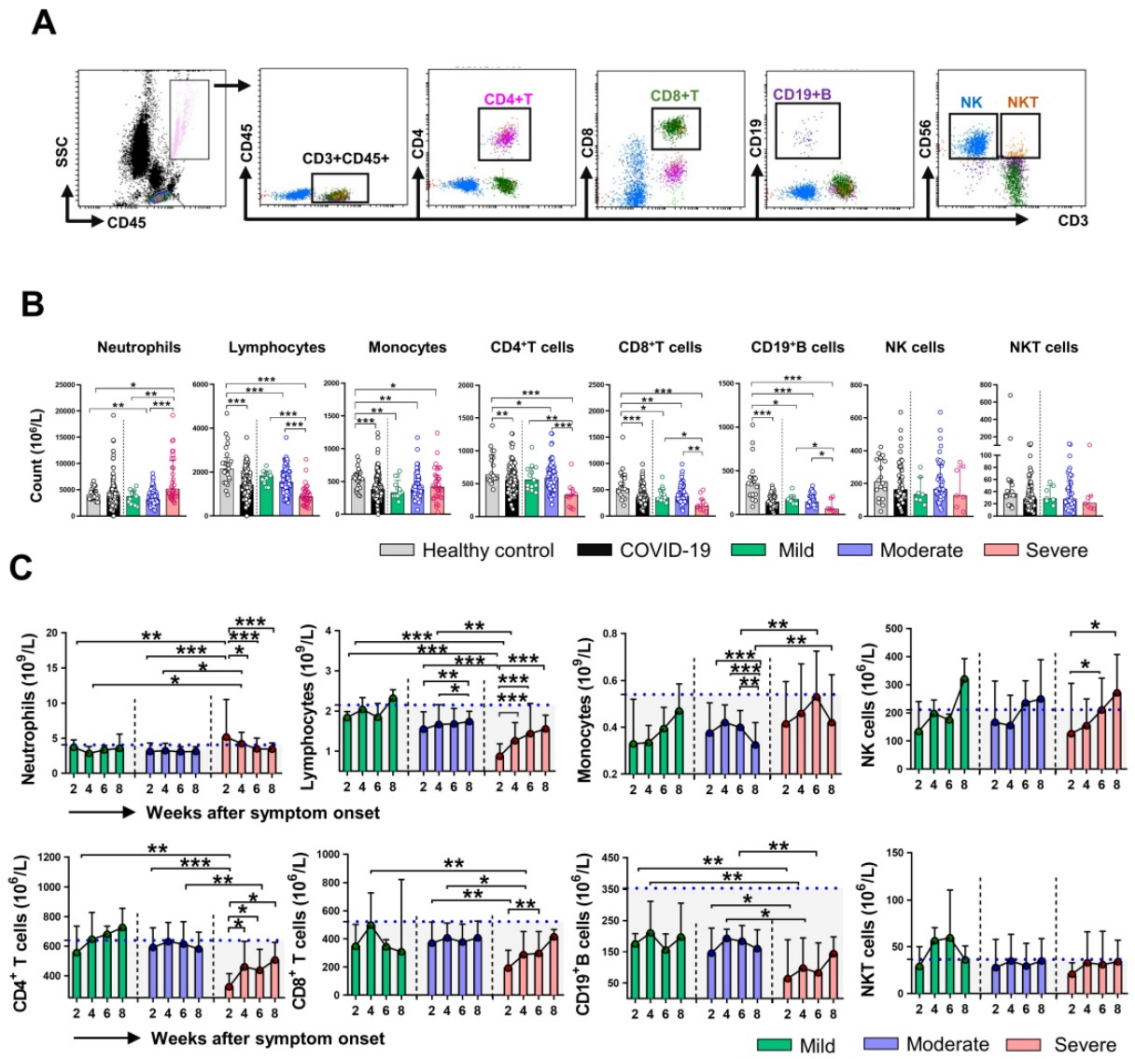
** Dynamic changes of immune cells count in COVID-19 patients.** (A) The representative gating strategy for CD4^+^ T, CD8^+^ T, CD19^+^ B, NK, and NKT cells. (B) The counts of neutrophils, lymphocytes, monocytes, CD4^+^ T, CD8^+^ T, CD19^+^ B, NK, and NKT cells in healthy control (n = 25), mild (n = 14), moderate (n = 110), and severe (n = 34) COVID-19 patients at the initial time point. (C) Dynamic change of counts of neutrophils, lymphocytes, monocytes, CD4^+^ T, CD8^+^ T, CD19^+^ B, NK, and NKT cells in classified patients with COVID-19. ^*^P < 0.05, ^**^P < 0.01, ^***^P < 0.001. The blue dashed lines represent the median value of each parameter of healthy control. Data are presented as median (interquartile range).

Firstly, we analyzed the absolute counts of different immune cell subsets in healthy control (HC) and COVID-19 patients at the initial time point (week 2). The gating strategy for the analysis is shown in Figure 2A. As shown in Figure 2B, the counts of lymphocytes, monocytes, CD4^+^ T cell, CD8^+^ T cell, and CD19^+^ B cell in COVID-19 patients were significantly lower than that in HC. Compared with the mild and moderate patients, severe patients showed reduced counts of lymphocytes, CD4^+^ T cell, CD8^+^ T cell, and CD19^+^ B cell, while increased counts of neutrophils. No significant differences in counts of monocytes, NK cells, NKT cells were observed among the classified COVID-19 patients. Next, we assessed the dynamic changes in cell counts among the three groups of COVID-19 patients. The absolute counts of lymphocytes, CD8^+^ T cell, and CD19^+^ B cell in severe patients were significantly lower than that in mild and moderate patients at week 2 and week 4; however, the cell counts were gradually increased and reached comparable levels to those in the mild and moderate patients at week 8. The differences in monocytes counts between the severe and moderate patients were significant at week 6 and week 8. We then investigated the dynamic profiles of cell counts in severe patients. A sustained decrease in neutrophils while an increase in lymphocytes counts was observed in the severe group during clinical observation. Of note, gradually increased counts of NK cells were observed in severe patients, and the differences in cell counts were significant at week 6 and week 8 compared with that at week 2 (Figure 2C). We also assessed the dynamic changes in the percentage of cell subsets. Not surprisingly, an increased percentage of neutrophils while a decreased percentage of lymphocytes was found in COVID-19 patients compare to that in HC (Figure S1A). The dynamic changes regarding the percentage of neutrophils and lymphocytes were similar to the findings in cell counts, and no significant differences in CD4^+^ T cell, CD19^+^ B cell, NK cell, and NKT cell percentage were observed among the three groups of COVID-19 patients during the whole course of the disease (Figure S1B).

### Dynamic expression of inflammatory cytokines in COVID-19 patients

**Figure 3:**
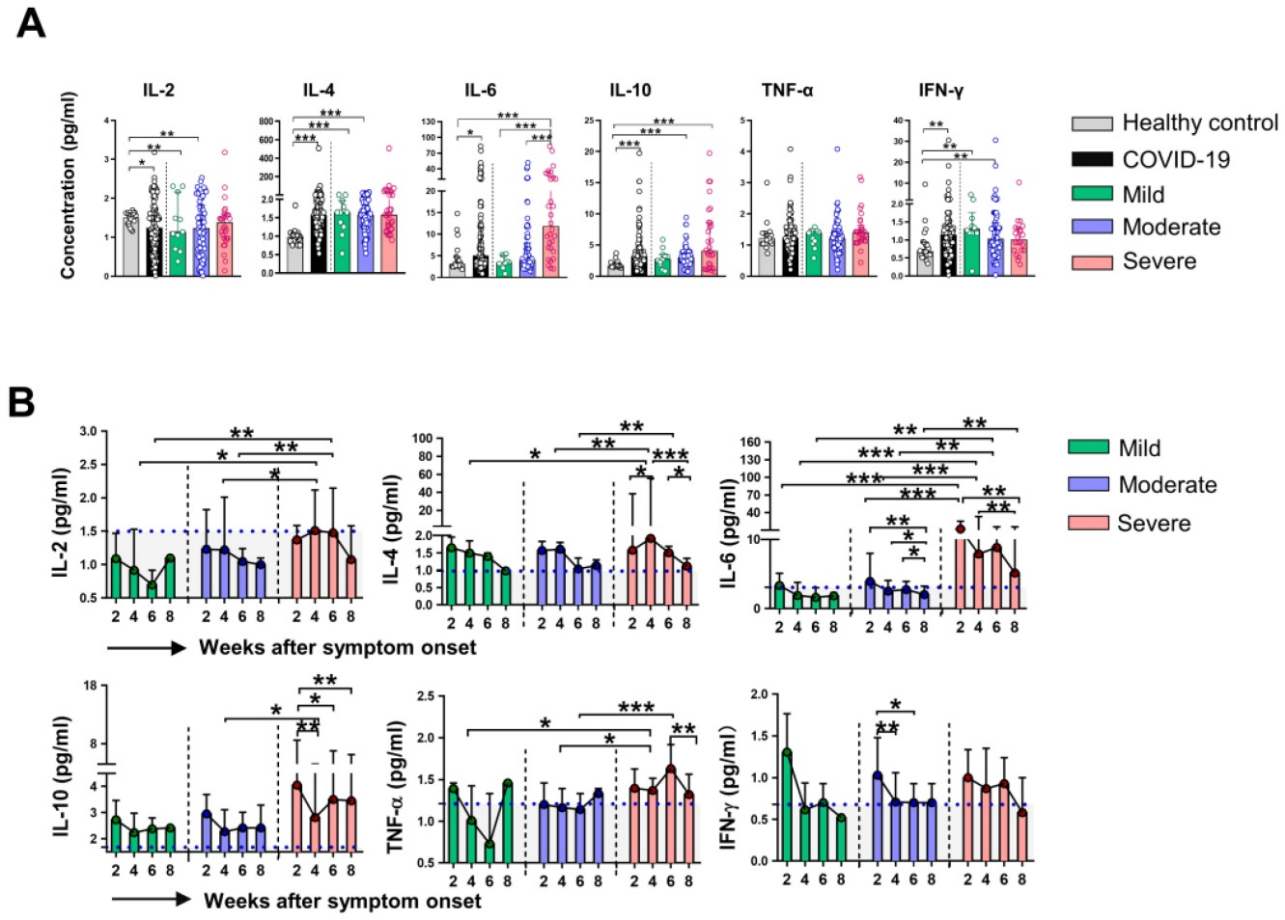
** Dynamic changes of cytokines in patients with COVID-19.** (A) The concentrations of IL-2, IL-4, IL-6, IL-10, TNF-α and IFN-γ in healthy control (n = 17) and patients with mild (n = 7), moderate (n = 48) and severe (n = 23) infection with SARS-CoV-2 at the initial time point. (B) Dynamic change of IL-2, IL-4, IL-6, IL-10, TNF-α, and IFN-γ in classified patients with COVID-19. ^*^P < 0.05, ^**^P < 0.01, ^***^P < 0.001. The blue dashed lines represent the median value of each parameter of healthy control. Data are presented as median (interquartile range).

We then assessed serum levels of inflammatory cytokines in HC and different groups of COVID-19 patients. Evaluation of cytokines at week 2 revealed that the levels of IL-4, IL-6, IL-10, and IFN-γ were higher, while the levels of IL-2 were lower in COVID-19 patients than that in HC. Notably, we found only dramatically elevated levels of IL-6 in severe patients compared with that in mild and moderate patients (Figure 3A), the levels of other indicated cytokines were comparable among the three groups of COVID-19 patients. We next examined the dynamic expression of cytokines in the classified COVID-19 patients. Elevated levels of IL-2, IL-4, IL-6, and TNF-α at week 4, as well as significant increases in IL-2 and IL-6 at week 6 were found in severe patients compared to that in mild and moderate patients. No significant difference in IFN-γ levels was observed among the three groups during the whole period of observation. Sequential detection showed a substantial decrease of IL-6 levels in moderate and severe patients, but IL-6 levels in severe patients were significantly higher than that in moderate patients at the corresponding time point (Figure 3B). We then analyzed whether immune cells and cytokines at the initial time point were associated with the severity of COVID-19. Univariable logistics regression analysis showed that variables with statistical significance including the counts of neutrophils, lymphocytes, CD4^+^ T cells, CD8^+^ T cells, CD 19^+^ B cells at week 2, as well as the levels of IL-2, IL-4, IL-6, and IL-10 at week 2 (Table S1); however, the multivariable analysis indicated that IL-6 was the only independent factor associated with the severity of COVID-19. Next, the ROC curve was drawn for the severity of COVID-19 based on the levels of IL-6. Results showed that the AUC of the ROC curve was 0.774 for IL-6 (*P* < 0.001), the optimal cut-off value for IL-6 concentration was 6.735 pg/mL, and the sensitivity and specificity to predict the severity of COVID-19 were 76.7% and 76.6%, respectively (Figure S2).

### Correlation between laboratory findings and clinical parameters in COVID-19 patients

**Figure 4:**
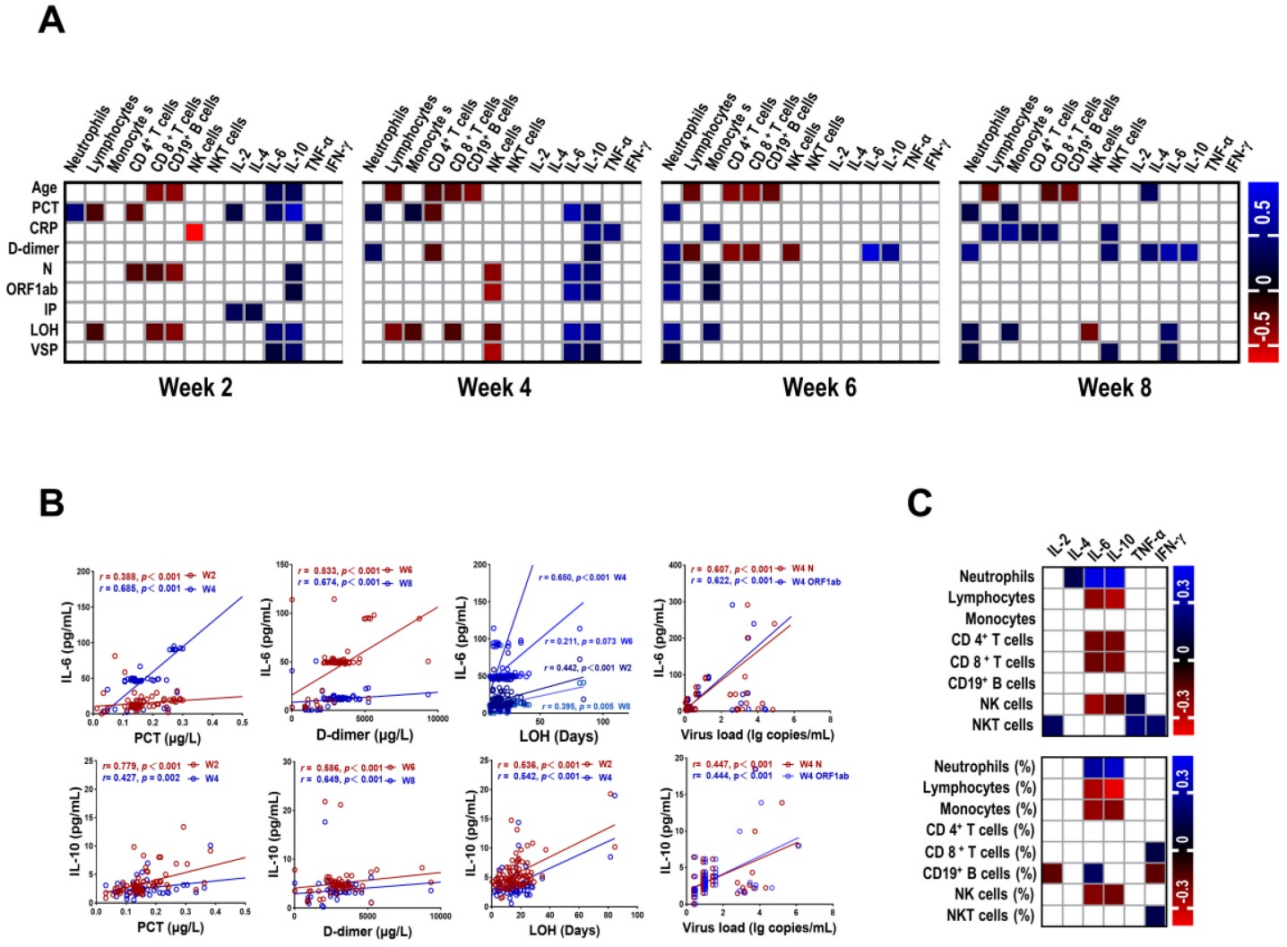
** Correlation between laboratory findings and clinical parameters in COVID-19 patients.** (A) Correlation between laboratory findings and clinical parameters in COVID-19 patients. (B) Scatter plot of correlation between laboratory findings (IL-6 and IL-10) and clinical parameters (PCT, D-dimer, LOH, and virus load) in COVID-19 patients. (C) Correlation between immune cells and cytokines in COVID-19 patients. Blanks in the figure denote no significant coefficient (*P* > 0.05). CRP, C-reactive protein; IP, incubation period; LOH, length of hospitalization; PCT, procalcitonin; VSP, virus shedding period; W, week.

We further investigated the relationship among the numbers of immune cells, the levels of cytokines and clinical parameters at different stages in patients with COVID-19. As showed in Figure 4A and Table S2 - S5, the majority of correlation coefficients were relatively low. Interestingly, IL-6 was positively correlated with PCT (*r* = 0.388, *P* < 0.001; *r* = 0.685, *P* < 0.001, respectively) and LOH (*r* = 0.442, *P* < 0.001; *r* = 0.650, *P* < 0.001, respectively) at week 2 and week 4. In addition, we also observed strong correlation between IL-6 and D-dimer at week 6 (*r* = 0.833, *P* < 0.001) and week 8 (*r* = 0.674, *P* < 0.001). Similar correlations were detected between IL-10 and PCT, LOH, D-dimer at corresponding time points (Figure 4B). Of note, IL-6 and IL-10 were correlated with the levels of N gene (*r* = 0.607, *P* < 0.001; *r* = 0.447, *P* < 0.001, respectively) and ORF1ab gene (*r* = 0.622, *P* < 0.001; *r* = 0.444, *P* < 0.001, respectively) of SARS-COV-2 at week 4. Besides, the number of neutrophils at week 6 was positively correlated with LOH (*r* = 0.551, *P* < 0.001), the levels of N and ORF1ab gene (*r* = 0.432, *P* < 0.001; *r* = 0.536, *P* < 0.001, respectively). Furthermore, we also found correlation between VSP and IL-6 at week 4 (*r* = 0.429, *P* = 0.001). In contrast, an inverse correlation was observed between NK cells and VSP at corresponding time (*r* = - 0.447, *P* = 0.001). We then analyzed the association between the numbers of immune cells and the levels of cytokines in COVID-19 patients. The preliminary analysis demonstrated a positive correlation between neutrophils and IL-6 and IL-10 (*r* = 0.449, *P* < 0.001; *r* = 0.469,* P* < 0.001, respectively) (Figure 4C, Table S6), suggesting a potential role of neutrophils in cytokine storm.

### Immune dysregulation in COVID-19 patients with persistent pulmonary injury

**Figure 5:**
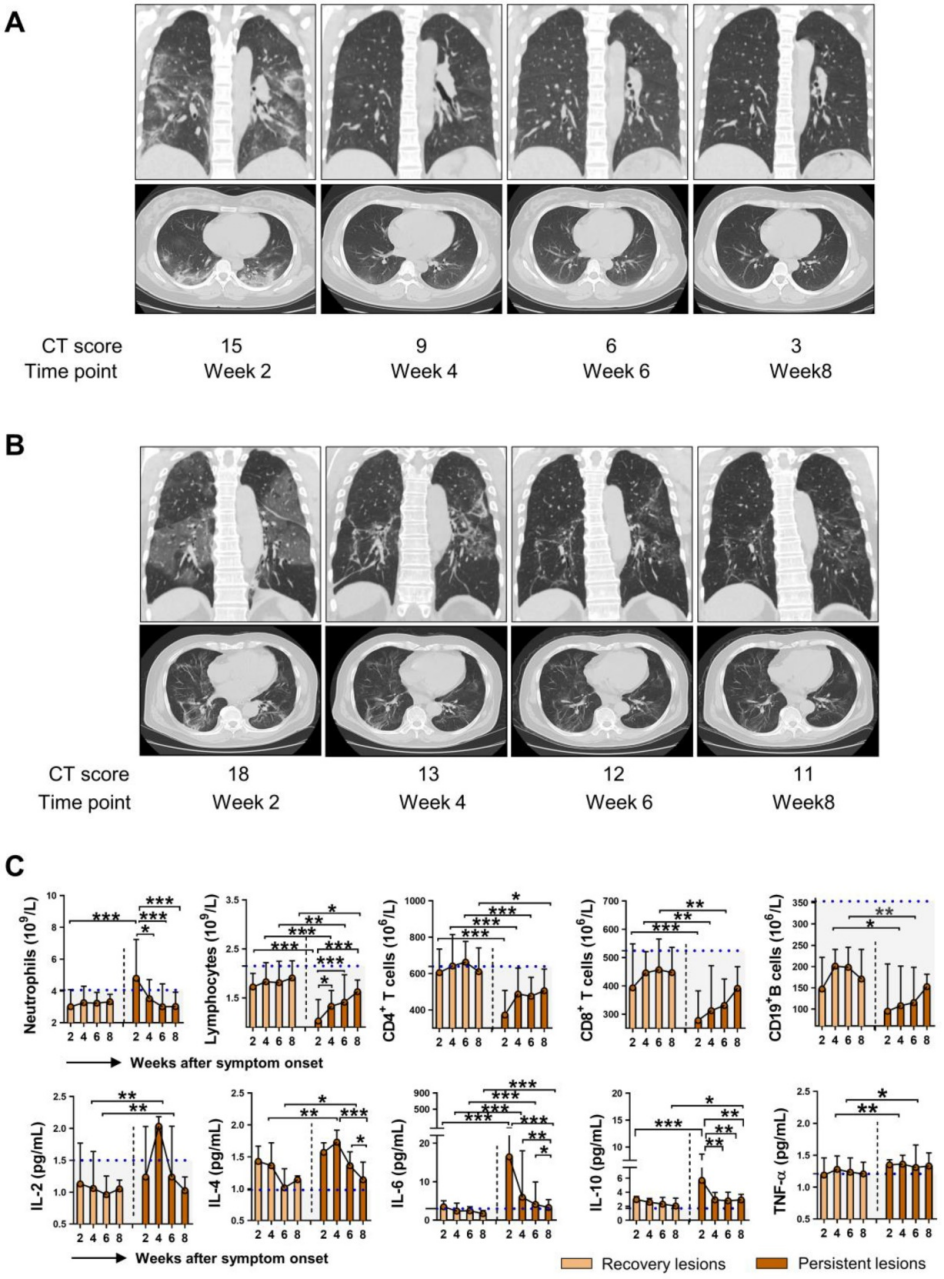
** The immunological features in COVID-19 patients with different outcomes of pulmonary injury.** Representative sequential chest CT images of lung window (coronal view and axial view) from patients with recovery lesions (A) and persistent lesions (B). The CT involvement score at the indicated time points was noted. (C) Dynamic changes of immune cells and cytokines in the patients with recovery lesions (n = 61) and persistent lesions (n = 49). The blue dashed lines represent the median value of each parameter of healthy control. ^*^P < 0.05, ^**^P < 0.01, ^***^P < 0.001. Data are presented as median (interquartile range). W, week.

As SARS-COV-2 mainly causes lung damage, we then investigated the influence of immune cells and cytokines on the outcome of pulmonary lesions in COVID-19 patients. Chest CT manifestations at week 8 were reviewed independently by 2 radiologists and were classified as recovery lesions (CT score < 5) and persistent lesions (CT score ≥ 5) based on the CT score. Figure 5A and 5B showed representative sequential chest CT images of lung window (coronal view and axial view) from patients with recovery lesions and persistent lesions, respectively. The CT involvement score at the indicated time points was also noted. We then further assessed the dynamic changes of immune cells and cytokines based on the outcome of lung lesions. As shown in Figure 5C, compared with patients with recovery lesions, patients with persistent lesions had significantly decreased numbers of lymphocytes, CD4^+^ T cells, CD8^+^ T cells, and CD19^+^ B cells, while markedly elevated levels of IL-6 at each indicated time point. Besides, patients with persistent lesions showed higher levels of IL-10 than that in patients with recovery lesions at week 2 and week 8. It should be noted that although a significantly increased number of lymphocytes and markedly decreased levels of IL-6 and IL-10 were observed in patients with persistent lesions during the disease course, the lymphocytes count did not reach to the median value of HC and the levels of IL-6 and IL-10 were consistently exceeded the median level of HC (blue dashed lines in Figure 5). Besides, similar changes regarding the percentage of neutrophils and lymphocytes were also observed in patients with persistent lesions; however, no significant differences in the percentage of CD4^+^ T cell, CD19^+^ B cell, NK cell, and NKT cell were observed between the two groups (Figure S3).

### IL-6 acts as a critical factor for pulmonary injury in COVID-19

**Figure 6:**
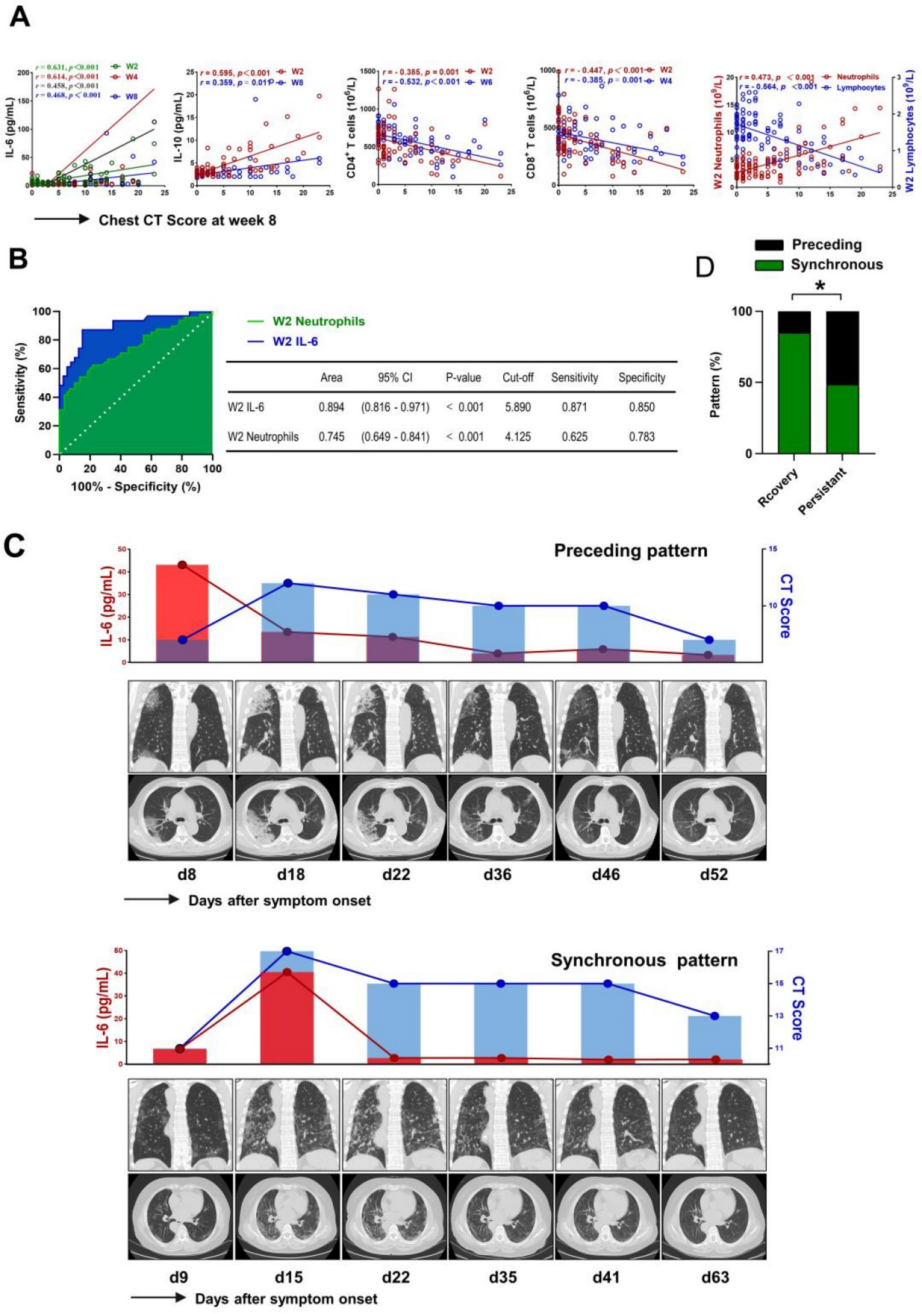
** The critical role of IL-6 in pulmonary injury.** (A) Scatter plot of correlation between laboratory findings (IL-6, IL-10, neutrophils, lymphocytes, CD4^+^ T cells, and CD8^+^ T cells) and chest CT score of week 8 in COVID-19 patients. (B) Receiver operating characteristic (ROC) curves were constructed, and the area under the ROC curves (AUC) was calculated to evaluate the predictive capability of IL-6 and neutrophils in identifying persistent pulmonary lesions of COVID-19 patients. (C) Representative sequential chest CT images of lung window (coronal view and axial view), serum levels of IL-6, and chest CT score in patients with preceding pattern (above) and non-preceding pattern (below). (D) The percentage of patients showing preceding or non-preceding pattern was compared between patients with recovery and persistent lesions. W, week.

We further investigated the association between the levels of IL-6 and pulmonary injury in patients with COVID-19. As shown in Figure 6A, the levels of IL-6 at each time point showed a positive correlation with chest CT score at week 8 (*r* = 0.631, *P* < 0.001; *r* = 0. 614, *P* < 0.001; *r* = 0. 458, *P* < 0.001; *r* = 0.468, *P* < 0.001, respectively). Meanwhile, we also found that the levels of IL-10 and the counts of neutrophils at week 2 were positively correlated with CT score (*r* = 0.595, *P* < 0.001; *r* = 0.473, *P* < 0.001, respectively). However, further analysis identified inverse correlation between lymphocytes, CD4^+^ T cell, CD8^+^ T cell at week 2 and CT score (*r* = - 0.564, *P* < 0.001; *r* = - 0.385, *P* = 0.001; *r* = - 0.447, *P* < 0.001, respectively) (Figure 6A). We then evaluated the association between laboratory findings and the outcome of pulmonary lesions using univariable logistics regression analysis. The preliminary analysis demonstrated that persistent pulmonary injury was associated with the counts of neutrophils, lymphocytes, CD4^+^ T cells, CD8^+^ T cells, and the levels of IL-6 and IL-10 at week 2 (Table S7). However, multivariable logistic regression analysis only confirmed the contribution of IL-6 (OR = 1.273 (1.068 - 1.518), *P* = 0.007) and neutrophils (OR = 1.975 (1.147 - 3.399), *P* = 0.014) in persistent pulmonary injury. Next, the ROC curves were drawn for the outcome of pulmonary lesions based on IL-6 levels and neutrophils counts. Results showed that the AUC of the ROC curve was 0.894 for IL-6 and 0.745 for neutrophils, respectively (*P* < 0.001). The optimal cut-off value for IL-6 concentration was 5.890 pg/mL, and the sensitivity and specificity were 87.1% and 85.0%, respectively; The optimal cut-off value for neutrophils counts was 4.125×10^9^/L, which gave a sensitivity for prediction of persistent lung injury of 62.5% and a specificity of 78.3%, respectively (Figure 6B). Next, we analyzed the temporal sequence between IL-6 and CT score in patients with different outcomes of pulmonary lesions. Peak expression of IL-6 before the maximum CT score was defined as a “preceding pattern,” by contrast, the other conditions, including concurrently boosted IL-6 and CT score, and peak level of IL-6 after the highest CT score, were defined as “non-preceding pattern” (Figure 6C). As shown in Figure 6D, the preceding pattern was found in 41.7% of patients with persistent lesions, but found in only 9.5% of patients with recovery lesions. The difference was significant between the two groups. This finding suggested that IL-6 might act as a risk factor leading to the aggravation of lung injury.

## Discussion

This study systematically depicted the longitudinal changes of the primary immune parameters and radiographic features with a sequential observation of COVID-19 patients. Our results showed that significant decreases in the counts of lymphocytes, especially CD4^+^ T cell, CD8^+^ T cell and B cell, and an increase in IL-6 levels are typically characterized by the severity of COVID-19 patients, indicating the dysregulated inflammation and antiviral immune responses may attribute to the development of SARS-COV-2 infection. In addition, the close relationship between low levels of IL-6 and favorable outcomes of pulmonary lesions suggested that a suitable application of therapy intervention targeting IL-6 may benefit the prognosis of the disease.

After the initial attacked of COVID-19, many clinical studies have improved the comprehension of its clinical features 1,18,19. The present study showed that the notable clinical manifestations in severe patients included fever, fatigue, diarrhea, and polypnea. Therefore, oxygen therapy, even mechanical ventilation, was commonly applied in critical patients. The previous study has noted a male predominance in the incidence of COVID-19; however, the number of male and female patients were comparable in our study, indicating that individuals are generally susceptible to SARS-CoV-2 infection. Consistent with a previous report 6, we also found that older patients, particularly those with underlying comorbidities, are more likely to develop severity of the disease, which suggested that SARS-CoV-2 is more likely to infect elderly individuals with chronic comorbidities due to weaker immune functions.

Lymphocytes are the most important immune cells to protect against viral infection 20-22. One of the typical hallmarks of SARS-CoV-2 infection is lymphopenia, which could be used as a reference index for the clinical diagnosis of SARS-CoV-2 infection 7. A recent study reported that SARS-CoV-2 infects T lymphocytes through its spike protein-mediated membrane fusion 23, which might be highly involved in the pathological process of SARS-CoV-2 infection. In the present study, we found that the development of lymphopenia in severe patients was related to the significantly decreased absolute counts of CD4^+^ T cell and CD8^+^ T cell and reduced counts of CD19^+^B cell, indicating SARS-CoV-2 may impair cellular and humoral immunity at an early stage. Of note, the numbers of lymphocytes were gradually increased in the severe patients during the course. The counts of CD4^+^ T cell, CD8^+^ T cell, and CD19^+^ B cell recovered to a comparable level to that of the mild patients after 4 weeks of the disease onset. Excluding 1 died patient; all the severe patients included in our study survived the disease; hence, we speculate that the restoration of lymphopenia is associated with the recovery of the disease. In addition, the alleviation of pulmonary lesions based on CT score is also closely correlated with the numbers of lymphocytes, this notion was also supported by the findings in which patients with a favorable outcome of lung lesions had relatively higher amounts of lymphocytes, CD4^+^ T cell, and CD19^+^ B cell compared with those with persistent injury. Different from that reported by Zheng *et al.*^24^, we did not observe decreased counts of NK cell lymphocytes in severe COVID-19 patients. Interestingly, we found increased counts of NK cells at the final observation time point compared with that at the initial stage. Actually, Zheng *et al.* also described recovered numbers of NK cells and decreased percentage of NKG2A^+^ NK cells in the convalescent period, suggesting a potential role of innate immunity in the control of SARS-CoV-2 infection.

The dampened adaptive responses lead to viral persistence and consequent “hyperinflammatory state” with aberrantly non-effective and ampliative innate immune. In consequence, the fulminating and continuous release of cytokines will attack COVID-19 patients swiftly and violently 25. Cytokines have been documented to play an essential role in immunopathology during coronavirus infections 26,27. Hyper-activation of the NF-κB pathway is involved in the process. One of the major pathways for NF-κB activation after coronavirus infection is the MyD88 pathway through pattern recognition receptors, which resulted in the generation of a variety of proinflammatory cytokines, including IL-6, TNF-α, and chemokines 4. Different from recent studies showing remarkably elevated levels of IL-6, IL-10, and IFN-γ in severe COVID-19 patients, we only observed notably increased levels of IL-6 in severe patients. IL-6 is considered as a critical factor in immune-related pneumonitis 28. Although IL-6 aids in the formation of follicular helper T cells and longevity plasm cells, it also actives PD-1, Tim-3, SOCS-3, downregulates the ability of dendritic cells to prime naïve T cells, induces rapid T cells exhaustion and impedes antiviral response during cytokine storm 28-30. Indeed, the ARDS seen with severe SARS-CoV-2 infection is a disorder induced by cytokine storms 31. However, the underlying molecular mechanism of the onset of the cytokine storm in COVID-19 patients is still elusive. Some explanations have been proposed. The high viral load of infected epithelial cells in the respiratory tract caused increased tissue damage and became a reservoir of proinflammatory cytokines^32^. In addition, a recent study reported that elevated glucose levels favored SARS-CoV-2 replication and cytokines production. Furthermore, SARS-CoV-2 infection-induced mtROS/HIF-1α, subsequently promoted glycolytic genes and cytokines in return. The exacerbated pro-inflammatory cytokines boosted the expression of PD-1 in CD4 T cells, induced T cells exhaustion and dysfunction. 33.

Although several studies have demonstrated an excellent discrimination ability of CT in COVID-19 severity, the established pulmonary lesions may influence the intervention for severe COVID-19 patients in early stages, and a reliable predictor is still urgently required 34,35. Of note, the dynamic analysis demonstrated that IL-6 levels are still maintained at a high level at the endpoint of observation in severe patients. In contrast, significantly decreased levels of IL-6 were found compared with that at the initial time point. Hence, efficient therapy was accompanied by reduced levels of IL-6. Indeed, we had no direct evidence for the involvement of IL-6 in lung pathology; however, we found that individuals with severe pulmonary lesions at the endpoint of observation showed high levels of IL-6 throughout the whole course, suggesting that duration of high IL-6 concentrations could be a risk factor for persistent pulmonary injury. More notably, we observed that the elevation of IL-6 before the aggravated pulmonary lesions in the majority of COVID-19 patients; thus, we speculated that IL-6 might serve as a driving factor for pulmonary pathogenesis during SARS-CoV-2 infection. Considering that the anti-IL-6R antibody (tocilizumab) is an effective treatment for cytokine release syndrome in CAR-T cell therapies 28,36, researchers might want to consider drugs with a similar mechanism of action in COVID-19 treatment. In fact, preliminary studies have confirmed the effectiveness of tocilizumab in severe COVID-19 patients 37-40, and a recent publication has demonstrated that impaired immune cell cytotoxicity in severe COVID-19 is IL-6 dependent 41, further supporting the hypothesis that targeting IL-6 signaling pathways could be a potential promise for severe and critical COVID-19 patients. Nevertheless, the appropriate time for an intervention of IL-6 warrants further investigation.

There were several limitations to our study that might cause some potential bias. Firstly, it was a retrospective, single-center study of patients admitted to the hospital, data at each observation time point were not available from all the subjects; standardized data from a prospective cohort would be better to assess the temporal change in patients with COVID-19. Secondly, this study only included a limited number of critical ill patients. A semi-quantitative analysis based on imagined involvement cannot fully reflect the grade of lung injury in such patients. Thirdly, we only assess the immune cells and cytokines from peripheral blood; the cells and cytokines derived from the pulmonary interstitium will further dissect the complex pathogenesis of SARS-CoV-2 infection.

## Conclusions

Our study demonstrated that the duration of high IL-6 concentrations could be a risk factor for persistent pulmonary injury during SARS-CoV-2 infection. Therefore, surveillance of IL-6 is helpful in the early screening and timely intervention for severe COVID-19 patients. Our work further supports the notion that IL-6 signaling pathways could be therapeutic targets to treat COVID-19.

## Supplementary Material

Supplementary figures and tables.

## Figures and Tables

**Table 1:** Clinical characteristics of patients with COVID-2019.

Variables	Mild patients (n = 14)	Moderate patients (n = 110)	Severe patients (n = 34)	*P* value
Age^&^	28.1 ± 11.9	46.0 ± 15.8	62.7 ± 14.2	<0.001
Male (%)	8/14 (57.1)	57/110 (51.8)	23/34 (67.6)	0.265
Smoking (%)	0/13 (0.0)	11/103 (10.7)	2/34 (5.9)	0.580
Comorbidities (%)	2/13 (15.4)	37/103 (35.9)	19/25 (76.0)	<0.001
Wuhan-contacted (%)	5/13 (38.5)	46/103 (44.7)	18/25 (72.0)	0.039
Incubation period^#^	10.0 (1.0 - 14.0)	7.0 (1.0 - 11.0)	4.0 (2.0 - 8.0)	0.260
**Symptoms**				
Fever (%)	2/13 (15.4)	67/103 (65.0)	32/34 (94.1)	<0.001
Dry cough (%)	3/13 (23.1)	31/103 (30.1)	11/34 (32.4)	0.844
Expectoration (%)	2/13 (15.4)	36/103 (35.0)	21/34 (61.8)	0.004
Sore throat (%)	0/13 (0.0)	17/103 (16.5)	3/34 (8.8)	0.203
Fatigue (%)	1/13 (7.7)	24/103 (23.3)	19/34 (55.9)	<0.001
Diarrhea (%)	0/13 (0.0)	9/103 (8.7)	9/34 (26.5)	0.013
Polypnea (%)	0/13 (0.0)	13/103 (12.6)	25/34 (73.5)	<0.001
Headache (%)	1/13 (7.7)	12/103 (11.7)	7/34 (20.6)	0.403
Chest pain (%)	0/13 (0.0)	2/102 (1.9)	2/34 (5.9)	0.485
Myalgia (%)	1/13 (7.7)	11/103 (10.7)	9/34 (26.5)	0.070
Anorexia (%)	1/13 (7.7)	15/103 (14.6)	15/34 (44.1)	0.001
Nausea (%)	1/13 (7.7)	3/103 (2.9)	3/34(8.8)	0.206
Vomiting (%)	0/13 (0.0)	3/103 (2.9)	1/34 (2.9)	1.000
**Laboratory findings**				
ALT*	23.7 (15.4 - 29.5)	19.9 (14.1 - 28.0)	23.0 (16.5 - 44.9)	0.146
AST*	19.7 (14.5 - 23.3)	18.7 (16.1 - 24.8)	29.9 (21.7 - 49.6)	<0.001
CK*	73.5 (48.5 - 129.0)	67.0 (47.5 - 116.0)	124.0 (57.5 - 209.0)	0.060
Creatinine*	64.0 (56.8 - 81.5)	61.8 (52.3 - 77.0)	66.7 (52.5 - 86.3)	0.396
CRP^§^	23.5 (23.5 - 23.5)	24.4 (17.4 - 36.8)	46.4 (29.0 - 78.6)	0.022
Procalcitonin^∆^	0.039 (0.026 - 0.059)	0.039 (0.030 - 0.056)	0.148 (0.068 - 0.260)	<0.001
D-dimer^∆^	900.0 (737.5 - 1187.5)	1030.0 (655.0 - 1317.5)	1640.0 (1090.0 - 2450.0)	<0.001
**Treatments**				
Antibiotic (%)	4/12 (33.3)	74/91 (81.3)	26/32 (81.3)	0.001
Antiviral (%)	2/12 (16.7)	42/91 (46.2)	23/32 (71.9)	0.002
Glucocorticoid (%)	0/12 (0.0)	14/91 (15.4)	20/32 (62.5)	<0.001
Oxygen therapy (%)	7/12 (58.3)	69/91 (75.8)	30/32 (93.8)	0.018
**Outcome**				
Discharge (%)	14/14 (100.0)	110/110 (100.0)	33/34 (97.1)	0.295
Hospitalization (%)	0/14 (0.0)	0/110 (0.0)	0/34 0.0)	
Death (%)	0/14 (0.0)	0/110 (0.0)	1/34 (2.9)	
Virus shedding period^#^	7.0 (5.0 - 14.0)	11.0 (8.0 -17.0)	14.5 (9.3 - 20.5)	0.023
Length of hospitalization^#^	11.0 (7.0 -19.0)	21.0 (14.0 - 28.0)	31.0 (22.5 - 35.0)	<0.001
				

Data are presented as median (interquartile range) or n/N (%), where N is the total number of patients with available data. *P* values comparing severe cases, moderate cases, and mild cases are from χ², Fisher's exact test, one-way ANOVA or Kruskal-Wallis H. ^&^Years;^ #^Days; *U/L, median (interquartile range); ^§^mg/L, median (interquartile range); ^∆^μg /L, median (interquartile range). ALT, alanine transaminase; AST, aspartate aminotransferase; CK, creatine kinase; CRP, C-reactive protein.

## Abbreviations

ARDS: acute respiratory distress syndrome
AUC: area under the curve
COVID-19: coronavirus disease 2019
CI: confidence interval
CT: computed tomographic
GGO: ground-glass opacities
HC: healthy control
IP: incubation period
LOH: length of hospitalization
MERS: middle east respiratory syndrome coronavirus
OR: odds ratio
ROC: receiver operating characteristic
SARS: severe acute respiratory syndrome coronavirus
SARS-CoV-2: severe acute respiratory syndrome coronavirus 2
VSP: virus shedding period
