# Supplementary material for: Longitudinal clinical and radiographic evaluation reveals interleukin-6 as an indicator of persistent pulmonary injury in COVID-19
Source: Int J Med Sci. 2021 Jan 1;18(1):29–41. doi: 10.7150/ijms.49728 (PMC7738960; doi:10.7150/ijms.49728)
Supplement: Supplementary file 1 — Supplementary figures and tables. [file ijmsv18p0029s1.pdf]

Supplementary Materials

Table S1. Univariable and multivariable analysis of factors at week 2 associated with the severity of COVID-2019.

Variables	Univariable analysis		Multivariable analysis	
	OR (95% CI)	*P* value	OR (95% CI)	*P* value
Neutrophils (10^9/L)	1.753 (1.373 - 2.238)	$<$0.001		
Lymphocytes (10^9/L)	0.058 (0.019 - 0.179)	$<$0.001		
$CD4^+$ T cells (10^6/L)	0.991 (0.986 - 0.997)	0.001		
$CD8^+$ T cells (10^6/L)	0.989 (0.983 - 0.996)	0.002		
$CD19^+$ B cells (10^6/L)	0.977 (0.959 - 0.996)	0.018		
IL-2 (pg/mL)	1.375 (0.773 - 2.445)	0.279		
IL-4 (pg/mL)	1.059 (1.016 - 1.103)	0.006		
IL-6 (pg/mL)	1.070 (1.026 - 1.115)	0.001	1.161 (1.034 - 1.303)	0.012
IL-10 (pg/mL)	1.317 (1.116 - 1.554)	0.001		

Table S2. Correlation between immunological features and clinical parameters at week 2 among COVID-2019 patients.

Variables		Age	PCT	CRP	D-dimer	N level	ORF1ab Level	IP	LOH	VSP	CT score
Neutrophils (count)	*r*		0.459								0.473
	P		< 0.001								< 0.001
Lymphocytes (count)	*r*		- 0.199						- 0.185		- 0.564
	P		0.029						0.029		< 0.001
Monocytes (count)	*r*										
	P										
$CD4^+$ T cells (count)	*r*		- 0.249			- 0.210					- 0.385
	P		0.028			0.048					0.001
$CD8^+$ T cells (count)	*r*	- 0.367				- 0.209			- 0.269		- 0.447
	P	< 0.001				0.049			0.011		< 0.001
CD 19^+ B cells (count)	*r*	- 0.379				- 0.339			- 0.348		
	P	0.007				0.017			0.014		
NK cells (count)	*r*			- 0.669							
	P			0.024							
NKT cells (count)	*r*										
	P										
Neutrophils (%)	*r*		0.322	0.304							0.645
	P		< 0.001	0.018							< 0.001
Lymphocytes	*r*		- 0.286	- 0.262							- 0.610

(%)	*P*		0.002	0.043							< 0.001
Monocytes (%)	*r*		- 0.254	- 0.334	0.232						- 0.353
	P		0.005	0.009	0.008						< 0.001
CD4$^+$ T cells (%)	*r*										
	P										
CD8$^+$ T cells (%)	*r*	-0.240									
	P	0.024									
CD 19$^+$ B cells (%)	*r*		- 0.388						- 0.346		
	P		0.011						0.015		
NK cells (%)	*r*							- 0.358			
	P							0.048			
NKT cells (%)	*r*	0.327	0.537						0.521	0.471	
	P	0.019	< 0.001						< 0.001	0.001	
IL-2 (pg/mL)	*r*	0.257						0.347			
	P	0.015						0.003			
IL-4 (pg/mL)	*r*							0.263			
	P							0.028			
IL-6 (pg/mL)	*r*	0.344	0.388						0.442	0.222	0.631
	P	0.001	< 0.001						< 0.001	0.036	< 0.001
IL-10 (pg/mL)	*r*	0.340	0.779			0.275	0.221		0.536	0.386	0.595
	P	0.001	< 0.001			0.009	0.038		< 0.001	< 0.001	< 0.001
TNF-α (pg/mL)	*r*			0.352							
	P			0.041							
IFN-γ (pg/mL)	*r*										
	P										

Blanks in this table denote no statistical significance ($P > 0.05$). CRP, C-reactive protein; IP, incubation period; LOH, length of hospitalization; PCT, Procalcitonin; VSP, virus shedding period.

Table S3. Correlation between immunological features and clinical parameters at week 4 among COVID-2019 patients.

Variables		Age	PCT	CRP	D-dimer	N level	ORF1ab Level	IP	LOH	VSP	CT score
Neutrophils (count)	*r*		0.270		0.347						0.228
	P		0.004		0.001						0.017
Lymphocytes (count)	*r*	- 0.255							- 0.331		- 0.427
	P	0.003							0.001		< 0.001
Monocytes (count)	*r*		0.220						- 0.207		0.235
	P		0.019						0.015		0.014
$CD4^+$ T cells (count)	*r*	- 0.210	- 0.230		- 0.218						- 0.359
	P	0.037	0.041		0.045						0.001
$CD8^+$ T cells (count)	*r*	- 0.255							- 0.197		- 0.385
	P	0.011							0.050		0.001
CD 19^+ B cells (count)	*r*	- 0.340									
	P	0.011									
NK cells (count)	*r*					- 0.362	- 0.424		- 0.268	- 0.447	
	P					0.007	0.001		0.048	0.001	
NKT cells (count)	*r*										
	P										
Neutrophils (%)	*r*	0.189	0.223		0.307				0.237		0.350
	P	0.027	0.017		0.001				0.005		< 0.001
Lymphocytes (%)	*r*	- 0.224	- 0.230		- 0.274				- 0.240		- 0.482
	P	0.008	0.014		0.002				0.005		< 0.001
Monocytes (%)	*r*				- 0.240	- 0.174			- 0.177		0.201
	P				0.007	0.041			0.037		0.036
$CD4^+$ T cells (%)	*r*							0.217			
	P							0.044			
$CD8^+$ T cells (%)	*r*		0.295								
	P		0.008								
CD 19^+ B cells (%)	*r*			0.624			0.269		0.301		
	P			0.007			0.047		0.025		
NK cells (%)	*r*					- 0.305	- 0.390			- 0.396	
	P					0.024	0.003			0.003	
NKT cells (%)	*r*				0.311						0.319
	P				0.028						0.037
IL-2 (pg/mL)	*r*										0.302
	P										0.028
IL-4 (pg/mL)	*r*										0.315
	P										0.021
IL-6 (pg/mL)	*r*		0.685			0.607	0.622		0.650	0.429	0.614
	P		< 0.001			< 0.001	< 0.001		< 0.001	0.001	< 0.001
IL-10 (pg/mL)	*r*		0.427	0.534	0.332	0.447	0.444		0.542	0.290	
	P		0.002	0.006	0.013	< 0.001	< 0.001		< 0.001	0.023	
TNF-α (pg/mL)	*r*			0.492							0.286
	P			0.012							0.038
IFN-γ (pg/mL)	*r*										
	P										

Blanks in this table denote no statistical significance (*P* > 0.05). CRP, C-reactive protein; IP, incubation period; LOH, length of hospitalization; PCT, Procalcitonin; VSP, virus shedding period.

Table S4. Correlation between immunological features and clinical parameters at week 6 among COVID-2019 patients.

Variables		Age	PCT	CRP	D-dimer	N level	ORF1ab Level	IP	LOH	VSP	CT score
Neutrophils (count)	*r*		0.480		0.528	0.432	0.536		0.551	0.311	
	P		< 0.001		< 0.001	< 0.001	< 0.001		< 0.001	0.001	
Lymphocytes (count)	*r*	- 0.272			- 0.237						- 0.275
	P	0.004			0.020						0.010
Monocytes (count)	*r*			0.457		0.240	0.253		0.358		0.311
	P			0.001		0.014	0.009		< 0.001		0.003
$CD4^+$ T cells (count)	*r*	- 0.350			- 0.348						- 0.532
	P	0.001			0.002						< 0.001
$CD8^+$ T cells (count)	*r*	- 0.372			- 0.310						- 0.389
	P	< 0.001			0.006						0.001
CD 19^+ B cells (count)	*r*	- 0.267									- 0.364
	P	0.021									0.003
NK cells (count)	*r*				- 0.283						
	P				0.019						
NKT cells (count)	*r*										
	P										
Neutrophils (%)	*r*		0.281		0.350	0.338	- 0.288		0.340		
	P		0.006		< 0.001	< 0.001	0.003		< 0.001		
Lymphocytes (%)	*r*	- 0.198	- 0.230		- 0.318				- 0.324		- 0.324
	P	0.039	0.027		0.001				0.001		0.002
Monocytes (%)	*r*				- 0.276	- 0.217	- 0.261		- 0.211	- 0.209	0.301
	P				0.006	0.026	0.007		0.027	0.030	0.005
$CD4^+$ T cells (%)	*r*										- 0.241
	P										0.040
$CD8^+$ T cells (%)	*r*										
	P										
CD 19^+ B cells (%)	*r*								0.228		
	P								0.049		
NK cells (%)	*r*	0.245								- 0.275	
	P	0.034								0.018	
NKT cells (%)	*r*									0.256	
	P									0.028	
IL-2 (pg/mL)	*r*										0.410
	P										0.001
IL-4 (pg/mL)	*r*										0.362
	P										0.004
IL-6 (pg/mL)	*r*				0.833						0.458
	P				< 0.001						< 0.001
IL-10 (pg/mL)	*r*				0.586						0.271
	P				< 0.001						0.033
TNF-α (pg/mL)	*r*										
	P										
IFN-γ (pg/mL)	*r*										
	P										

Blanks in this table denote no statistical significance ($P > 0.05$). CRP, C-reactive protein; IP, incubation period; LOH, length of hospitalization; PCT, Procalcitonin; VSP, virus shedding period.

Table S5. Correlation between immunological features and clinical parameters at week 8 among COVID-2019 patients.

Variables		Age	PCT	CRP	D-dimer	N level	ORF1ab Level	IP	LOH	VSP	CT score
Neutrophils (count)	*r*		0.265		0.534				0.274	0.242	
	P		0.024		< 0.001				0.010	0.024	
Lymphocytes (count)	*r*	- 0.262		0.453							- 0.333
	P	0.014		0.002							0.004
Monocytes (count)	*r*		0.315	0.562					0.277		0.277
	P		0.007	< 0.001					0.009		0.016
$CD4^+$ T cells (count)	*r*			0.348							- 0.286
	P			0.030							0.019
$CD8^+$ T cells (count)	*r*	- 0.223		0.394							
	P	0.050		0.013							
CD 19^+ B cells (count)	*r*	- 0.299									
	P	0.041									
NK cells (count)	*r*								- 0.299		
	P								0.043		
NKT cells (count)	*r*			0.413	0.410					0.291	
	P			0.023	0.010					0.049	
Neutrophils (%)	*r*				0.231						
	P				0.043						
Lymphocytes (%)	*r*		- 0.254								- 0.315
	P		0.031								0.006
Monocytes (%)	*r*		0.238	0.327							0.327
	P		0.044	0.030							0.004
$CD4^+$ T cells (%)	*r*									0.244	
	P									0.032	
$CD8^+$ T cells (%)	*r*										
	P										
CD 19^+ B cells (%)	*r*										
	P										
NK cells (%)	*r*	0.391							- 0.306	- 0.419	
	P	0.007							0.039	0.004	
NKT cells (%)	*r*				0.561					0.348	
	P				< 0.001					0.018	
IL-2 (pg/mL)	*r*										
	P										
IL-4 (pg/mL)	*r*	0.355			0.431						
	P	0.012			0.006						
IL-6 (pg/mL)	*r*				0.674				0.395	0.287	0.468
	P				< 0.001				0.005	0.045	< 0.001
IL-10 (pg/mL)	*r*				0.649						0.359
	P				< 0.001						0.011
TNF-α (pg/mL)	*r*										
	P										
IFN-γ (pg/mL)	*r*										
	P										

Blanks in this table denote no statistical significance (P > 0.05). CRP, C-reactive protein; IP, incubation period; LOH, length of hospitalization; PCT, Procalcitonin; VSP, virus shedding period.

Table S6. Correlation between immune cells and cytokines in patients with COVID-2019.

Variables		IL-2	IL-4	IL-6	IL-10	TNF-α	IFN-γ
Neutrophils (count)	*r*		0.153	0.449	0.469		
	P		0.013	< 0.001	< 0.001		
Lymphocytes (count)	*r*			- 0.226	- 0.282		
	P			< 0.001	< 0.001		
Monocytes (count)	*r*						
	P						
$CD4^+$ T cells (count)	*r*			- 0.185	- 0.188		
	P			0.002	0.004		
$CD8^+$ T cells (count)	*r*			- 0.174	- 0.194		
	P			0.004	0.003		
CD 19^+ B cells (count)	*r*						
	P						
NK cells (count)	*r*			- 0.244	- 0.154	0.153	
	P			0.001	0.031	0.033	
NKT cells (count)	*r*	0.188				0.247	0.207
	P	0.009				0.001	0.004
Neutrophils (%)	*r*			0.312	0.398		
	P			< 0.001	< 0.001		
Lymphocytes (%)	*r*			- 0.273	- 0.362		
	P			< 0.001	< 0.001		
Monocytes (%)	*r*			- 0.235	- 0.210		
	P			< 0.001	0.001		
$CD4^+$ T cells (%)	*r*						
	P						
$CD8^+$ T cells (%)	*r*						0.132
	P						0.044
CD 19^+ B cells (%)	*r*	- 0.147		0.190			- 0.148
	P	0.040		0.007			0.039
NK cells (%)	*r*			- 0.209	- 0.181		
	P			0.003	0.011		
NKT cells (%)	*r*						0.144
	P						0.045

Blanks in this table denote no statistical significance ($P > 0.05$).

Table S7. Univariable and multivariable analysis of factors at week 2 associated with chest CT score of the final time point.

Variables	Univariable analysis		Multivariable analysis	
	OR (95% CI)	*P* value	OR (95% CI)	*P* value
Neutrophils (10^9/L)	1.685 (1.286 - 2.206)	< 0.001	1.975 (1.147 - 3.399)	0.014
Lymphocytes (10^9/L)	0.077 (0.028 - 0.214)	< 0.001		
$CD4^+$ T cells (10^6/L)	0.994 (0.991 - 0.998)	0.002		
$CD8^+$ T cells (10^6/L)	0.993 (0.989 - 0.998)	0.003		
$CD19^+$ B cells (10^6/L)	0.988 (0.976 - 1.000)	0.058		
IL-2 (pg/mL)	1.444 (0.789 - 2.640)	0.223		
IL-4 (pg/mL)	1.623 (0.545 - 4.837)	0.385		
IL-6 (pg/mL)	1.314 (1.131 - 1.526)	< 0.001	1.273 (1.068 - 1.518)	0.007
IL-10 (pg/mL)	2.447 (1.445 - 4.141)	0.001		

Supplementary figure and figure legends

Figure S1

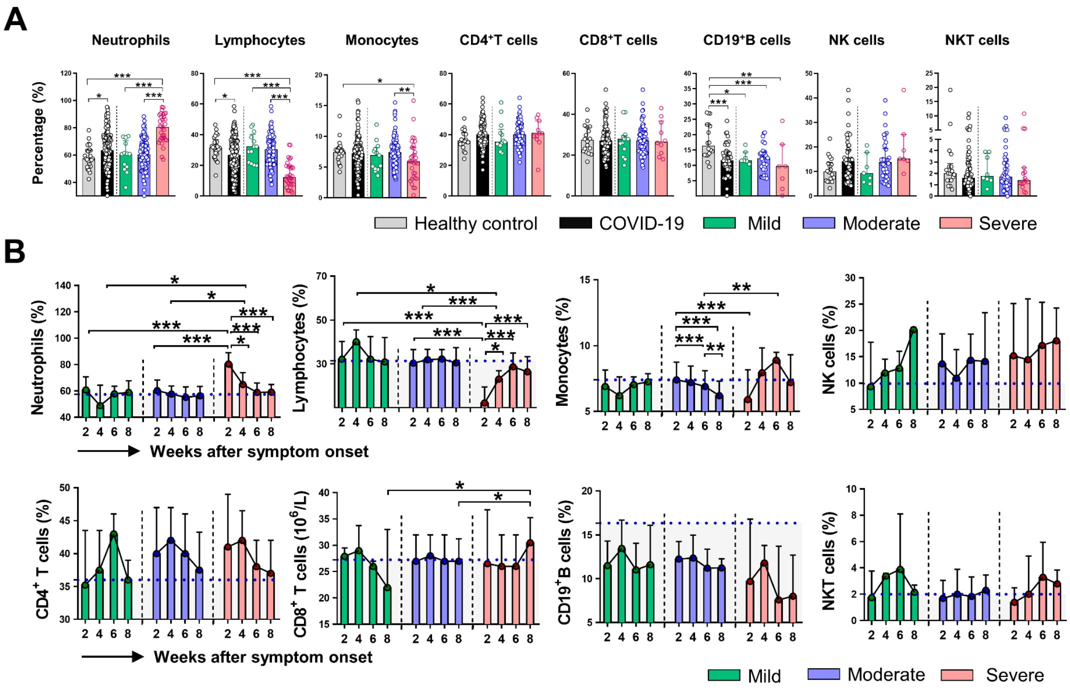

Figure S1. Dynamic changes of the percentage of immune cells in patients with COVID-19. (A) The percentage of neutrophils, lymphocytes, monocytes, $CD4^+$ T, $CD8^+$ T, $CD19^+$ B, NK, and NKT cells in healthy control (n = 25) and patients with mild (n = 13), moderate (n = 102) and severe (n = 25) infection with SARS-CoV-2 at the initial time point. (B) Dynamic change of percentage of neutrophils, lymphocytes, monocytes, $CD4^+$ T, $CD8^+$ T, $CD19^+$ B, NK, and NKT cells in patients with COVID-19. $^{*}P < 0.05$, $^{**}P < 0.01$, $^{***}P < 0.001$. Data are presented as the median (interquartile range).

Figure S2

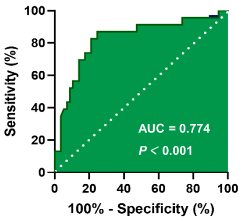

	Area	95% CI	P-value	Cut-off	Sensitivity	Specificity
Disease Severity	0.774	(0.670 - 0.878)	< 0.001	6.735	0.767	0.766

Figure S2. A receiver operating characteristic (ROC) curve was constructed, and the area under the ROC curves (AUC) was calculated to evaluate the predictive capability of IL-6 in identifying the severity of COVID-19.

Figure S3

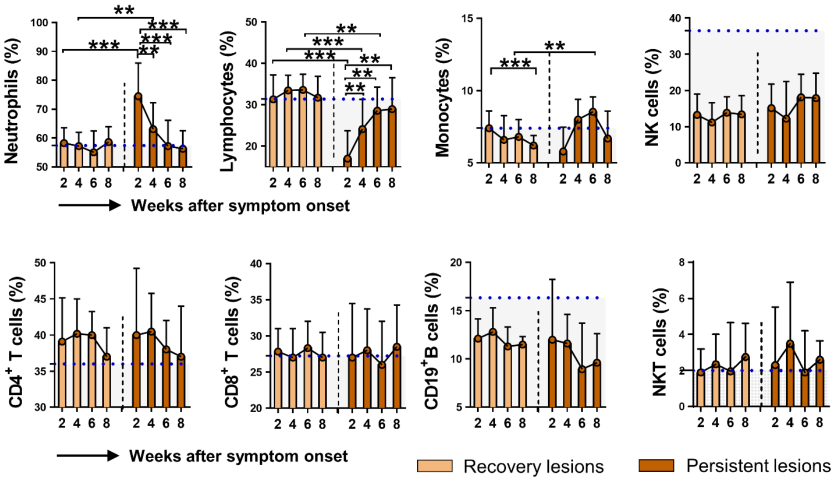

Figure S3. Dynamic changes of percentage of neutrophils, lymphocytes, monocytes, $CD4^+$ T, $CD8^+$ T, $CD19^+$ B, NK, and NKT cells in COVID-19 patients with recovery lesions and persistent lesions. $^{*}P < 0.05$, $^{**}P < 0.01$, $^{***}P < 0.001$. Data are presented as the median (interquartile range).
